# Genomic selection and reproducibility: are complex models distracting us from true scientific validity in the presence of genotype-by-environment interaction?

**DOI:** 10.1093/g3journal/jkaf244

**Published:** 2025-11-03

**Authors:** Réka Howard, Alexander E Lipka

**Affiliations:** Department of Statistics, University of Nebraska–Lincoln, Lincoln, NE 68583, United States; Department of Crop Sciences, University of Illinois at Urbana-Champaign, Urbana, IL 61801, United States

**Keywords:** genomic prediction, genomic selection, reproducibility

## Abstract

Genomic selection is poised to remain an exemplary plant breeding tool if reproducibility is maximized. Defined as the consistency of results when using the same data and approaches, reproducibility is as crucial as predictive ability when assessing the performance of genomic selection. Striving for reproducibility instead of model complexity will bolster efforts to make genomic selection applicable to more situations, as well as understandable to a wider range of end-users. The promises of high-dimensional, multi-environment data to contribute to accurate genomic-estimated breeding values are more likely to be attained if reproducibility is maximized. A focus on reproducibility will underscore trust among plant breeders and other practitioners using genomic selection.

Genomic selection has revolutionized plant breeding by dramatically accelerating the pace of genetic improvement. By utilizing genome-wide markers, it allows for accurate prediction of cultivars’ genetic potential, even for traits that are challenging or time-consuming to assess. This rapid evaluation bypasses the need for lengthy phenotypic trials, enabling breeders to quickly identify and select superior individuals, ultimately leading to faster genetic gains and more efficient breeding cycles. Building upon the foundational work of Bernardo (1994) and the groundbreaking paper by Meuwissen et al. (2001), the field of genomic selection has seen substantial development. While reproducibility is undeniably important in this field, we contend that the current focus often prioritizes computational consistency over true scientific validity and practical utility. Indeed, we argue that the community's emphasis on reproducibility should shift to recognize that simpler, conceptually sound models are frequently superior to highly complex ones, particularly when the latter yield only marginal predictive ability gains at a significant cost to interpretability, reproducibility, and genuine replicability across diverse breeding scenarios.

In this paper, we focus on reproducibility, i.e. the consistency of results using the same data, analysis methods, and computational tools (Lin 2020). A critical aspect is often overlooked, which is the robustness of the underlying statistical model's fundamental assumptions and its conceptual alignment with the problem, which dictates true scientific validity beyond mere computational consistency. In the modeling context, robustness refers to the extent to which a model can maintain consistent and reliable performance when exposed to new, noisy, or altered data or data differing from the training set. Validity, on the other hand, pertains to how accurately a model's predictions or inferences represent the underlying biological or breeding processes of interest. A model might be computationally reproducible yet still lack validity if it is based on flawed assumptions that do not align with biological reality. Similarly, a model may lack robustness if its predictive performance significantly declines when applied to different or slightly modified datasets. Within the context of genomic selection, reproducibility is the ability to consistently obtain similar genomic prediction accuracies and breeding value estimates when applying the same genomic prediction model and repeating the analysis on the same dataset. Reproducibility is important in genomic selection because it ensures the reliability and robustness of prediction models, fostering trust in their application to breeding programs. Furthermore, consistent results validate the accuracy and generalizability of genomic selection methods. Inaccurate or unreproducible predictions can have a negative impact and can lead to substantial economic losses and delays in genetic improvement; thus, it is important to consider reproducibility in genomic selection studies. Furthermore, breeders relying solely on highly complex models without the robust fundamental conceptual framework may constitute misrepresenting results.

While a complex model might be computationally reproducible, demonstrating consistent performance on a single dataset, its predictive power can be highly specific to that dataset's nuances. This raises a crucial concern: Complicated models, meticulously tuned for particular scenarios, may fail to generalize effectively to new or slightly different breeding environments. Consequently, prioritizing simpler, more interpretable models such as genomic best linear unbiased prediciton (GBLUP) over some of the machine learning techniques, even if they yield a few percentage points less accuracy on a specific training set, often provides greater practical value and true robustness for real-world breeding programs, as their underlying assumptions are more likely to hold across a broader range of conditions. However, it is important to recognize that simplicity alone does not guarantee robustness or validity. A simpler model, if poorly specified or trained on biased or nonrepresentative data, can be just as misleading as a complex model. For instance, if environmental covariates or genotype-by-environment interactions (G × E) are ignored in favor of simplicity, the model may systematically fail to generalize across diverse conditions. Thus, a model's interpretability and parsimony must be balanced with a careful examination of its assumptions, training context, and alignment with the biological question. Without this, neither robustness nor validity can be assured—regardless of model complexity.

The perceived appeal of marginal gains in prediction accuracy from highly complex models must be weighed against the tangible costs of their deployment, including increased computational resources, specialized expertise for implementation and maintenance, and the greater difficulty in diagnosing and explaining prediction failures to breeders and other end users. Moreover, the “black box” nature of some of these intricate models can create a significant communication gap between model developers and plant breeders, hindering the practical adoption and effective utilization of genomic selection in routine decision-making processes.

To maximize the impact of genomic selection on both basic and applied science, two major areas involving reproducibility need to be addressed. The first area involves the data used in genomic selection. The inclusion of improved and more diverse information into prediction models has the potential to increase prediction accuracy in genomic prediction models if they can explain phenotypic variability. However, it is important to consider how this information is included. Traditionally, genomic selection methods are trained using either best linear unbiased estimations (BLUEs) or best linear unbiased predictions (BLUPs) of genotypes as the response variable instead of the “raw” phenotypes at each individual environment (as described in, e.g. Piepho et al. 2008). Researchers refer to this as a two-stage analysis. These BLUEs and BLUPs account for the experimental design where the training set was obtained from. However, the assumption that a simple description of the experimental design adequately conveys the details of the BLUE/BLUP calculations, while model specifications remain ambiguous, presents a significant vulnerability to the reliability of genomic selection outcomes, especially when dealing with the complexities of unbalanced data.

The availability of improved high-density SNP arrays and whole-genome sequencing data has provided comprehensive genomic information. These data have the potential to significantly enhance the accuracy of genomic selection models by capturing a greater proportion of the genetic variation influencing complex traits. However, variations in SNP array platforms, genotyping protocols, and data processing pipelines can introduce inconsistencies in SNP values, leading to potential reproducibility issues in genomic selection models. At the SNP data processing stage, the variation in missing SNP value imputation can result in different predictions due to different imputation techniques (e.g. mean imputation, k-nearest neighbors, and software-specific imputation) altering the genetic relationship captured by the SNP data. Also, standardization methods (e.g. centering and scaling) can affect the magnitude of SNP effects in the model. The persistent ambiguity of these quality control specifications, particularly concerning minor allele frequency and missingness thresholds, introduces a level of subjective interpretation, which directly contradicts the fundamental principles of scientific rigor and reproducibility of genomic selection studies.

High-throughput phenotyping technologies (Cabrera-Bosquet et al. 2012) (e.g. remote sensing utilizing drones and satellites, automated imaging with spectral cameras, and sensor-based field monitoring) have allowed for more accurate and efficient measurement of complex traits, such as plant height, biomass, disease severity, and yield components. When applied to genomic selection, the availability of such high-throughput data allows for making selections based on optimal genomic estimated breeding values during multiple time points throughout the field season. The absence of community-wide documentation of standards and protocols for high-throughput data processing, particularly in image analysis and software validation, ignores the significant economic and scientific costs of irreproducible results.

Similarly, data from different -omics layers (e.g. transcriptomics, metabolomics, and proteomics) have made it possible to quantify the functional information of intermediate layers between observed genotypes and phenotypes. These -omics data have been incorporated into genomic selection models as both additional response variables and additional predictors (e.g. Schrag et al. 2018). Although extremely beneficial, these additional data present significant reproducibility challenges. Variations in platforms, protocols, and normalization methods across -omics layers can introduce inconsistencies. The high dimensionality and complexity of -omics data, coupled with challenges in annotation and computational workflows, further complicate reproducibility.

Traditional genomic selection models consider data from a single or limited number of environments. This can lead to biased predictions and suboptimal performance when such genomic selection models are applied to new or contrasting environments. By integrating data from a wide range of environments, genomic selection models can capture the G × E effects that significantly influence complex traits. Incorporating the G × E into genomic selection enables the identification of genotypes with broad adaptation or those specifically suited to specific environments. Incorporating weather covariates such as temperatures, rainfall patterns, soil types, and management practices can contribute to a more precise and more detailed characterization of the environments and thus a better understanding of G × E. Historic weather covariates add a valuable temporal dimension to genomic selection by modeling how certain genotypes responded past environmental scenarios. Historic data enable the inclusion of both long-term environmental trends and current cyclic patterns. This information could assist with the identification of genotypes with consistent performance across diverse climatic conditions. Other than the issues mentioned for the other data types (e.g. how standardization and missingness are handled), depending on where the weather data are obtained from (e.g. weather stations, satellite, and climate models), the data sources can lead to inconsistencies in the environmental covariates used in genomic selection models.

The second area where a focus on reproducibility could underscore genomic selection is with methodological advancements. Machine learning (ML) and artificial intelligence (AI) approaches offer a powerful avenue for capturing nonlinear relationships and intricate interactions that traditional linear models often overlook (Lourenco et al. 2024). These approaches can potentially extract hidden patterns in genetic architecture without explicitly modeling the patterns. This could result in improved predictive ability, particularly for complex traits influenced by numerous genetic and environmental factors and their interactions (i.e. G × E). Simultaneously, refining Bayesian models through the exploration of different prior distributions allows for a more accurate representation of prior knowledge and uncertainty, leading to more robust and reliable predictions. This ability to consider different prior distributions is crucial for incorporating diverse data sources and accounting for varying levels of confidence in prior information.

However, the inherent “black box” nature of some ML, AI, and Bayesian algorithms can make it difficult to trace the decision-making process. This hinders reproducibility because it makes the task of replicating and interpreting results more difficult. Variations in hyperparameter tuning, network architecture, or training protocols can lead to substantial differences in model performance, even with identical datasets. Similarly, the flexibility of Bayesian models, while beneficial for incorporating prior knowledge, can lead to discrepancies if prior specifications are not clearly documented or if different researchers make subjective choices. Furthermore, the integration of diverse data sources may compound these issues. It is crucial to recognize that while complex ML/AI models might yield marginal gains, perhaps only a few percentage points, over simpler and more transparent methods such as GBLUP, these improvements do not always justify the increased complexity, computational burden, and diminished interpretability. Often, simpler models provide a more robust and generalizable solution across varied scenarios, making them superior for practical breeding program implementation. Without standardized workflows, transparent documentation of model parameters and tuning, and readily available code, the reproducibility of genomic selection studies employing these advanced techniques can become problematic. Also, addressing the “black box” problem through methods that enhance model interpretability and explainability is paramount, not only to ensure reproducibility but also to foster trust and facilitate the broader adoption of these powerful tools in breeding programs.

Incorporating the wide availability of high-throughput genotyping, phenotyping, multiomics, and environmental information into genomic selection presents unique sets of challenges. Combining these different types of data is not trivial. The significant differences in data sizes among these types of data could result in the models not taking potentially small data sizes into consideration. Various algorithms exist for integrating multiomics data, each with its own limitations and assumptions. Moreover, the integration of multiple data types involves complex computational workflows that could be reliant on specific versions of software tools and libraries. Variations in software versions, operating systems, or computational environments can lead to inconsistencies in the results. Given the inherent complexity and potential for variability in multidata integration, it is essential that we prioritize the development and dissemination of these powerful approaches through free and open-source software. By providing access to these tools, we not only enable researchers to reproduce and validate results but also foster a more inclusive and collaborative scientific community. This ensures that the benefits of cutting-edge genomic selection methodologies are available to a broader audience, thereby accelerating innovation and enhancing the impact of genomic selection on global agriculture.

Moreover, the importance of training set optimization is underscored for multienvironment genomic selection studies. With strategies such as sparse testing to maximize information content and minimize bias, we can ensure that genomic selection models are robust, accurate, and applicable across a wide range of breeding scenarios. Variations in sparse testing methodologies, such as the specific criteria used for selecting individuals or the algorithms employed, can lead to different training sets and consequently, different prediction performances. Furthermore, the inherent stochasticity of sampling strategies can lead to inconsistencies across studies. Without rigorously standardized protocols for training set construction and detailed documentation of the underlying assumptions and parameters, replicating the composition of the training set becomes challenging. It is also important to point out that protocols might provide a framework, but they are often insufficient—especially when they are utilized for a different data/situation—without a transparent record of the *why* behind each decision. The selection of specific individuals, the application of filtering criteria, and the handling of potential biases are all driven by assumptions, and if they are not addressed, they can make replication impossible.

It is well documented that the practical implementation of genomic selection models into breeding programs resulted in accelerated genetic progress in many crops. However, this notable success falls outside the scope of this article, which is primarily concerned with the reproducibility of genomic selection studies related to data and modeling. Genomic selection has been executed in numerous plant breeding programs including major crops such as maize, soybean, wheat, rice, cotton, and sorghum. With the implementation of genomic selection into their programs, plant breeders can improve traits such as yield, disease resistance, stress tolerance, grain and other quality traits, and drought tolerance. However, the successful integration of genomic selection into these diverse breeding programs depends on the reproducibility of the underlying predictions. This is directly tied to data integrity and modeling robustness. Ensuring that predictions are consistent and replicable across different datasets and models is critical for building confidence in genomic selection as a tool for sustainable agricultural improvement. While the pursuit of the highest possible predictive ability is tempting, the practical value of genomic selection in real-world breeding programs is contingent not just on raw performance numbers, but on the trustworthiness and generalizability of those predictions across varied and unforeseen conditions. Therefore, focusing exclusively on complex machine learning models that offer only marginal gains over simpler, more interpretable alternatives such as GBLUP may divert resources and attention from factors that truly enhance long-term genetic progress and the reliable deployment of these technologies. Ultimately, enhanced reproducibility will solidify genomic selection's role in driving genetic gain and contributing to global food security, allowing breeders to confidently deploy these powerful tools to develop resilient and high-performing varieties.

While genomic selection has undeniably transformed plant breeding, its continued success and widespread adoption depend heavily on addressing the critical issue of reproducibility. We highlighted key areas where reproducibility challenges arise, primarily focusing on data-related issues and modeling methodologies. The increasing complexity of data, from high-throughput phenotyping and multiomics integration to environmental covariates, demands stringent standardization and transparent documentation at every stage of data processing and analysis, including justifications for certain decisions. Similarly, the implementation of advanced ML, AI, and Bayesian modeling techniques necessitates clear reporting of model parameters, tuning strategies, and computational workflows to ensure reproducibility. Crucially, we argue that the community should refine its focus on reproducibility to prioritize scientific validity and practical utility, recognizing that simpler, conceptually sound models may often be superior to highly complex ones that offer only marginal gains in predictive ability at the cost of interpretability and genuine replicability across diverse scenarios. However, this preference for simplicity must be tempered with a critical evaluation of whether a model is truly valid and robust. As genomic selection continues to evolve, a commitment to rigorous reproducibility practices will be essential for realizing its full potential in addressing global food security and sustainable agriculture.
